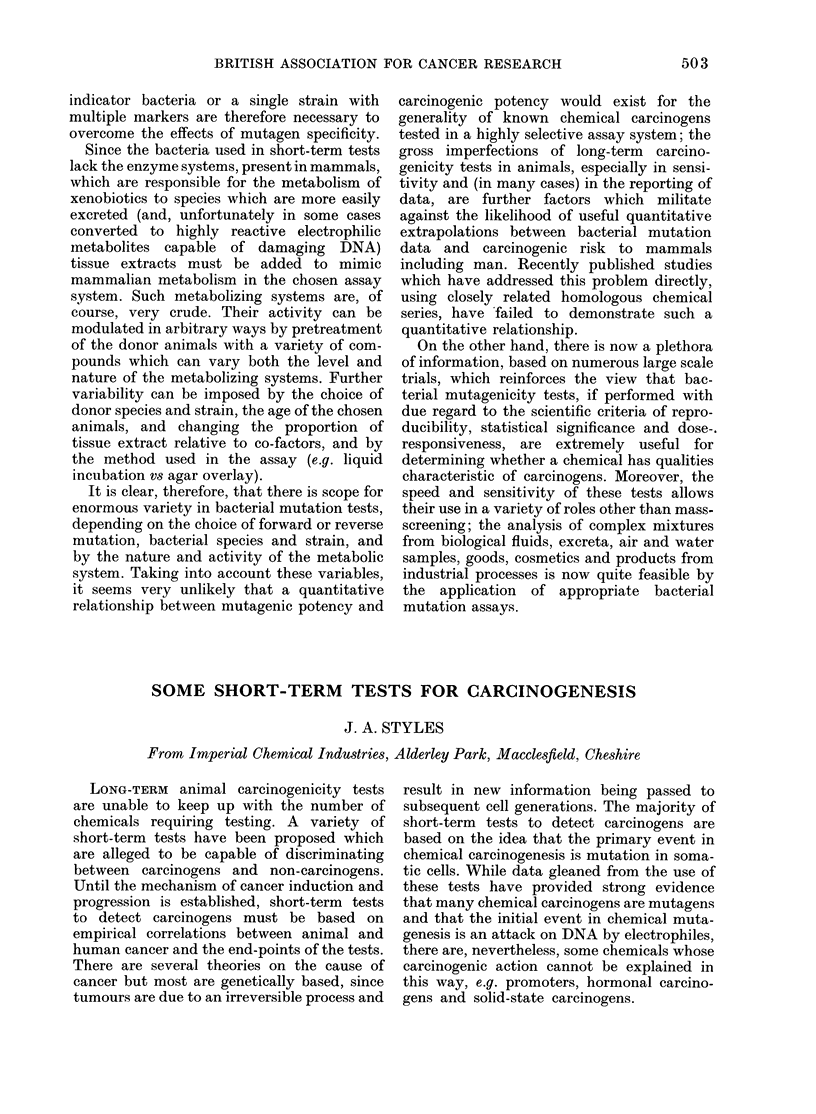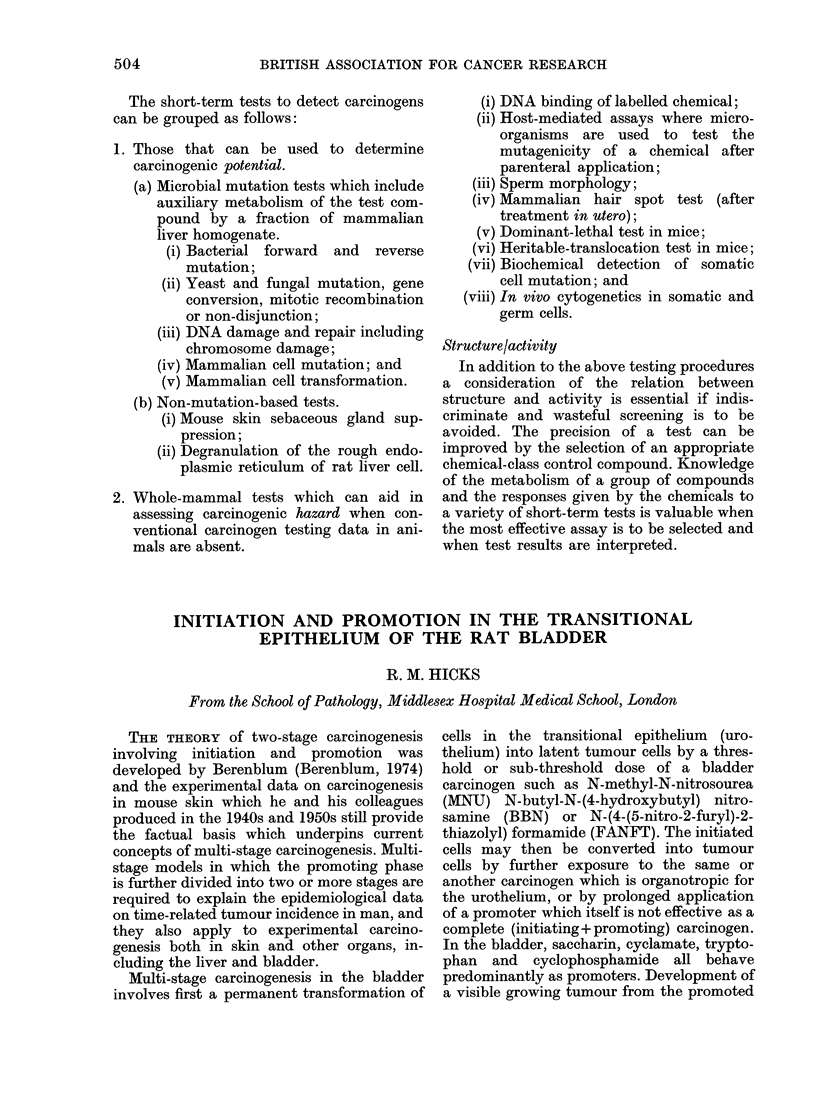# Some short-term tests for carcinogenesis.

**DOI:** 10.1038/bjc.1980.83

**Published:** 1980-03

**Authors:** J. A. Styles


### SOME SHORT-TERM TESTS FOR CARCINOGENESIS

J. A. STYLES

*From Imperial Chemical Industries, Alderley Park, Macclesfield, Cheshire*

LONG-TERM animal carcinogenicity tests are unable to keep up with the number of chemicals requiring testing. A variety of short-term tests have been proposed which are alleged to be capable of discriminating between carcinogens and non-carcinogens. Until the mechanism of cancer induction and progression is established, short-term tests to detect carcinogens must be based on empirical correlations between animal and human cancer and the end-points of the tests. There are several theories on the cause of cancer but most are genetically based, since tumours are due to an irreversible process and result in new information being passed to subsequent cell generations. The majority of short-term tests to detect carcinogens are based on the idea that the primary event in chemical carcinogenesis is mutation in somatic cells. While data gleaned from the use of these tests have provided strong evidence that many chemical carcinogens are mutagens and that the initial event in chemical mutagenesis is an attack on DNA by electrophiles, there are, nevertheless, some chemicals whose carcinogenic action cannot be explained in this way, *e.g.* promoters, hormonal carcinogens and solid-state carcinogens.

The short-term tests to detect carcinogens can be grouped as follows:

1. Those that can be used to determine carcinogenic *potential.*
   (a) Microbial mutation tests which include auxiliary metabolism of the test compound by a fraction of mammalian liver homogenate.
      (i) Bacterial forward and reverse mutation;
      (ii) Yeast and fungal mutation, gene conversion, mitotic recombination or non-disjunction;
      (iii) DNA damage and repair including chromosome damage;
      (iv) Mammalian cell mutation; and
      (v) Mammalian cell transformation.
   (b) Non-mutation-based tests.
      (i) Mouse skin sebaceous gland suppression;
      (ii) Degranulation of the rough endoplasmic reticulum of rat liver cell.
2. Whole-mammal tests which can aid in assessing carcinogenic *hazard* when conventional carcinogen testing data in animals are absent.
   (i) DNA binding of labelled chemical;
   (ii) Host-mediated assays where micro-organisms are used to test the mutagenicity of a chemical after parenteral application;
   (iii) Sperm morphology;
   (iv) Mammalian hair spot test (after treatment *in utero*);
   (v) Dominant-lethal test in mice;
   (vi) Heritable-translocation test in mice;
   (vii) Biochemical detection of somatic cell mutation; and
   (viii) *In vivo* cytogenetics in somatic and germ cells.

*Structure/activity*

In addition to the above testing procedures a consideration of the relation between structure and activity is essential if indiscriminate and wasteful screening is to be avoided. The precision of a test can be improved by the selection of an appropriate chemical-class control compound. Knowledge of the metabolism of a group of compounds and the responses given by the chemicals to a variety of short-term tests is valuable when the most effective assay is to be selected and when test results are interpreted.